# Cardiomyocyte proliferation in zebrafish and mammals: lessons for human disease

**DOI:** 10.1007/s00018-016-2404-x

**Published:** 2016-11-03

**Authors:** Gianfranco Matrone, Carl S. Tucker, Martin A. Denvir

**Affiliations:** 1 The University of Edinburgh/British Heart Foundation Centre for Cardiovascular Science, The Queen’s Medical Research Institute, The University of Edinburgh, Edinburgh, EH16 4TJ Scotland; 2 Department of Cardiovascular Sciences, Houston Methodist Research Institute, Houston, 77030 TX USA

**Keywords:** Zebrafish, Mammals, Heart, Proliferation, Regeneration

## Abstract

Cardiomyocytes proliferate profusely during early development and for a brief period after birth in mammals. Within a month after birth, this proliferative capability is dramatically reduced in mammals unlike lower vertebrates where it persists into adult life. The zebrafish, for example, retains the ability to regenerate the apex of the heart following resection by a mechanism predominantly driven by cardiomyocyte proliferation. Differences in proliferative capacity of cardiomyocytes in adulthood between mammals and lower vertebrates are closely liked to ontogenetic or phylogenetic factors. Elucidation of these factors has the potential to provide enormous benefits if they lead to the development of therapeutic strategies that facilitate cardiomyocyte proliferation. In this review, we highlight the differences between Mammalian and Zebrafish cardiomyocytes, which could explain at least in part the different proliferative capacities in these two species. We discuss the advantages of the zebrafish as a model of cardiomyocyte proliferation, particularly at the embryonic stage. We also identify a number of key molecular pathways with potential to reveal key steps in switching cardiomyocytes from a quiescent to a proliferative phenotype.

**Electronic supplementary material:**

The online version of this article (doi:10.1007/s00018-016-2404-x) contains supplementary material, which is available to authorized users.

## Introduction

Ischaemic heart disease is the commonest cause of heart failure in developed countries either as a consequence of acute myocardial infarction or chronic ischaemic damage [1]. In addition the impact of an ageing population with degenerative cardiac disease associated with hypertension or valvular heart disease also contributes to cardiomyocyte dysfunction, loss and ultimately heart failure [2–4]. In the face of injury and progressive loss of cardiomyocytes, the human heart typically responds with hypertrophy combined with hyperplasia of non-cardiomyocyte cell populations in the heart including fibroblasts [5].

Embryonic and fetal hearts of most vertebrates, including mammals, typically show some degree of regenerative capacity, although studies in neonatal mice suggest it can last for up to 7 days after birth [6]. The limited capacity of the adult mammalian heart to restore lost cardiomyocytes following injury contrasts starkly with the highly effective regenerative process in adult hearts from lower vertebrates including amphibians and fish. Recently, the underlying mechanisms of heart regeneration in the adult zebrafish have become clearer with evidence that mature cardiomyocytes dedifferentiate, re-enter the cell cycle and then proliferate to support repair and replacement of injured myocardium [7, 8]. The zebrafish embryo has also contributed significantly to elucidate mechanisms of cardiac growth and development [9]. Despite being a two-chambered organ, the zebrafish heart exhibits many similarities to the mammalian heart and cellular and molecular studies clearly illustrate the common evolutionary origin of these structures [10]. Since the regenerative process appears to reactivate developmental pathways [11], understanding zebrafish cardiac development also helps us to understand the powerful cardiac regenerative ability in the adult zebrafish heart. A key question is whether a better understanding of the zebrafish cardiac regenerative process provides important insights into the mammalian response to injury and repair, and it remains an exciting challenge to determine if the regenerative capacity of the mammalian heart could be harnessed as a therapeutic strategy with significant clinical value.

There is no doubt, however, that the zebrafish represents an interesting model in which to study cardiomyocyte proliferation and cardiomyocyte hyperplasia following injury. One major advantage of utilizing zebrafish embryos for studies of cardiogenesis is that the embryo is able to survive for several days without a functioning cardiovascular system. This permits the study of cardiac phenotypes that would otherwise be lethal in mammalian model systems [12]. This review examines the differences and similarities in the response of mammals and zebrafish to cardiac injury. We also highlight several unique properties of the zebrafish embryo as a model of cardiomyocyte proliferation.

## Cardiomyocyte proliferation: from early embryos to adulthood

### Embryonic development

The heart is the first organ to visibly form and function during embryogenesis [13]. In Vertebrates, cardiogenesis is a morphologically complex process that involves sequential heart primordia migration, folding, looping, septation and maturation to form the chambered heart. The following description of heart development applies mainly to the mammalian heart although many aspects also apply to the zebrafish, and chicken heart. During embryogenesis, proliferation of new cardiomyocytes is the main source for the heart growth. Cardiomyocytes proliferate along the heart tube walls and within the atrioventricular septum. In particular, the outer surface of the heart, also called the compact region [14], achieves the highest proliferative rate. The epicardium, the thin layer of cells enveloping the heart, provides a source of mitogenic signaling that stimulate proliferation of cardiomyocytes within the compact zone [15]. A key epicardial-derived regulator of cardiac growth includes retinoic acid and its related receptors [16]. Newly formed cardiomyocytes thicken the ventricular wall and organize fingerlike projections along the inner ventricle surface giving rise to trabeculae, structures that increase force of contraction and improve oxygen and nutrient exchange for the heart itself. The endocardium, the specialized single-cell inner layer of the heart, also provides fundamental growth signals for embryonic cardiomyocytes, including peptides of the neuregulin family and their related tyrosine kinase receptors [17]. These growth factors also play an important role in promoting the normal “ballooning” of the outer curvature of the ventricle [18]. The intense cardiomyocyte proliferative activity observed during embryonic heart growth is accompanied by increasing intra-cavity shear forces that contribute to the shaping of the early heart [19].

### Fetal to adulthood transition

While cardiomyocytes divide extensively and rapidly during fetal life, in mammals they lose their proliferative capacity shortly after birth. The proliferative activity of murine cardiomyocytes starts to decrease around E10–12 [20]. One key molecular player at this stage is Jumonji (*jarid2*) that acts to inhibit cardiomyocyte proliferation through repression of cyclin D1 expression. Jumonji appears to repress cyclin D1 transcription by recruiting histone H3–K9 methyltransferases, G9a and GLP, to the cyclin D1 promoter [21]. Indeed, *jarid2* mutant mice demonstrate increased proliferation and overexpression of cyclin D1 in cardiomyocytes at E10 [22] [20].

In humans, cardiomyocyte proliferative capacity is lost by a few months after birth when cardiomyocytes withdraw from the cell cycle and remain in G0 stage, apparently indefinitely [23–26], a process called terminal differentiation. Downregulation of several fetal genes and upregulation of genes responsible for the adult phenotype play important roles in this process. Cyclin-dependent kinases (CDKs) play a core functional role in the cell cycle machinery. Sequential activation of different CDKs, forming complexes with their specific cyclins, allows progression of the cell cycle. In mammals, CDK4/6–cyclin D is activated in phase G1, CDK2–cyclin E in phase G1/S, CDK2/1–cyclin A in phase S/G2 and CDK1–cyclin B in phase M. Diminished CDK activity leads to attenuation or cessation of the cell cycle. The expression and activity of many cyclins and CDKs change synchronously during embryonic and postnatal developmental stages [27], suggesting a highly orchestrated series of cellular mechanisms controlling their role in proliferation. CDKs are regulated by CDK inhibitors (CKIs) including the INK4 family (p15, p16, p18 and p19) and Cip/Kip family (p21, p27 and p57) [27]. CKIs participate in termination of postnatal mammalian cardiomyocyte cell cycle as demonstrated in p21 and p27 knockout mice, where cardiomyocytes exit the cell cycle at G1-phase [28]. Meis1 is a transcriptional factor that is known to activate p21 and regulate cardiac cell cycle exit [29]. In fact, cardiomyocyte proliferative activity is prolonged after birth in Meis1 KO mice.

**Fig. 1:**
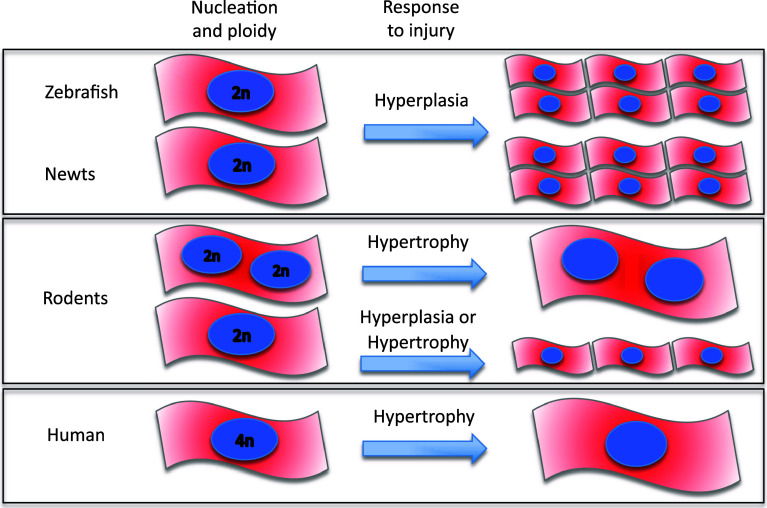
Cardiomyocyte cellular structure across species. Zebrafish and newt are mostly mononucleated and diploid [59, 154]; an organization that seems to favour a higher proliferative response to injury. Rodents show either mono- or bi-nucleated diploid cardiomyocytes [38]. Following stress or injury, mostly, these cardiomyocytes respond with hypertrophy; however, only those mononucleated cells appear to initiate proliferation. Human cardiomyocytes are mostly mononucleated and tetraploids. Limited data in young humans up to 20 years old, suggest that cardiomyocytes have some proliferative capacity [51, 52]. However, in later life, hypertrophy is the predominant response to injury in human

The cardiomyocyte cell cycle appears closely coupled to the accumulation of cell mass during development which acts to maintain consistent cell size [30]. In most species, this transition from hyperplastic-to-hypertrophic activity is characterized by changes in degree of ploidy and number of nuclei as cardiomyocytes undergo additional DNA replication followed by cytokinesis and/or karyokinesis [31, 32] (Fig. 1). The higher number of mononucleated and diploid cardiomyocytes in species capable of cardiac regeneration, such as newt [33], zebrafish [34] and rodent fetal and neonates [6, 35–37], suggest a higher proliferation capacity in such cells.

Indeed, Bersell et al. [38] demonstrated that only mononucleated cardiomyocytes that respond to the activation of the neuregulin 1/ErbB4 pathway after cardiac injury in mice initiate cardiomyocyte proliferation. Adult human cardiomyocytes are mostly mononucleated and tetraploid (4n), and adult mice cardiomyocytes are mainly binucleated and diploid (2n) [39] (Fig. 1). Indeed, several reports have shown a gradual decrease in the incorporation of radiolabeled thymidine soon after birth, coinciding with the formation of binucleated cardiomyocytes in mice [35, 40]. This process is associated with an increase in myofibril density and the formation of mature intercalated discs [41].

Fetal to adulthood temporal transition represents the divide between cardiac regenerating and non-regenerating species and it is, therefore, a key stage in which to study differences in cell cycle exit between species.

### Adulthood

During the twentieth century, it was believed that the heart is a post-mitotic organ and cardiac growth in the adult was attributed exclusively to cardiomyocyte hypertrophy [42–45]. However, since the 1990s, evidence of cardiomyocyte proliferation in adult human hearts has been gradually accepted [46–48]. Quaini and co-workers demonstrated the presence of proliferating cell nuclear antigen, a marker of the G1–S cell cycle phases, in adult human hearts with ischemic and dilated cardiomyopathy. Evidence of metaphasic chromosomes together with cytokinesis was demonstrated in normal myocardium and in ischemic and dilated cardiomyopathy and in myocardial infarction [46, 47]. Considerable disagreement remains on the frequency of these cellular events in adult normal and diseased adult myocardium [49]; however, there appears to be a clear cardiac cellular response to disease and injury [39, 50]. The measured rate of cell division is influenced by the methods used to detect DNA synthesis and count cardiomyocytes [35, 50]. Radiocarbon birth dating has shown that cardiomyocyte turnover is approximately 1% over 20–40-year-old hearts [51]. A more recent study using new confocal imaging and optical dissector method suggested that cardiomyocyte division contributes to heart growth in young humans up to 20 years of age, with a cardiomyocyte turnover of approximately 1.9% [52]. Some reports have shown that adult rat cardiomyocytes can proliferate in culture after treatment with FGF1 concomitant with p38MAP kinase inhibition [25]. Indeed, normal adult cardiomyocytes rarely appear to re-enter the cell cycle and proliferate [41], and terminally differentiated mammalian cardiomyocytes show a predominantly hypertrophic response to mitogenic stimuli [53, 54]. One significant difference between embryonic and adult heart is the expression of cell cycle promoters, such as CDKs, cyclins, and proto-oncogenes, markedly expressed in embryonic hearts, corresponding to high cardiomyocyte cell cycle activity, while their expression is lower in the adult heart. In general, negative cell cycle regulatory genes, such as CDK inhibitors, are upregulated in adult hearts, where cardiomyocyte cell cycle activity is extremely low [41].

There is now a well-recognized link between mammalian cardiomyocyte hypertrophy and proliferation which involves a complex series of interconnected signaling pathways, including JAK, PLC, JNK, ERK, calcineurin, STAT, RAS, and MEF2 [5]. In addition to an increase in cellular RNA and protein, hypertrophy results in transcriptional reprogramming that closely resembles the fetal gene program that is known to drive hyperplasia in the developing fetus [55, 56]. It is hypothesized that cardiomyocyte hypertrophy, without hyperplasia, in mammals might be the result of a fundamental block in karyokinesis and cytokinesis by which the adult cardiomyocyte is unable to disassemble sarcomeres, uncouple from neighbouring cells and divide [57].

### Mammals and zebrafish

Compared to mammals, cardiomyocytes from lower vertebrates, including teleost fish and salamander, show high proliferative capacity in adulthood [58]. The molecular mechanisms underlying this trait are not well understood. Better understanding could potentially provide important therapeutic targets for a range of cardiac disorders where cardiomyocyte loss plays a major role. A relevant difference between mammalians and zebrafish is that, in the latter, cardiomyocytes do not undergo cytokinetic mitosis. In fact, the majority of cardiomyocytes in adult zebrafish continue to have a single nucleus and a diploid genome (2*n*), similar to that observed in fetal mammalian hearts (vide supra) and associated with significant proliferative ability [59]. There is a common set of genes that drive growth and development of the embryonic heart in mammals [60] and fish [61]. A similar gene program is activated in the adult mammalian heart following injury or haemodynamic stress [5, 62, 63]. Many of these genes also appear to be activated in response to resection of the ventricle apex in the zebrafish [8, 64]. While this process has been studied in a variety of mammalian animal models, less is known about the related processes in the adult human heart.

Becker and colleagues [65] created and analysed a transgenic zebrafish embryo carrying a mutation in the troponin (*tnnt2*) gene associated with hypertrophic cardiomyopathy (HCM) in humans. An important finding in this model was that although the mutant gene resulted in abnormal sarcomeric organization, similar to that observed in humans and mouse models with HCM, the zebrafish exhibited a hyperplastic rather than a hypertrophic phenotype. Thus, the response of the zebrafish to stress or injury appears to be, primarily, a hyperplastic response, in contrast to mammals where the response is almost exclusively one of hypertrophy.

## The heart’s response to injury: hypertrophy versus hyperplasia

### In mammals

A variety of mechanisms are present in all organisms for dealing with tissue damage resulting from injury or disease. Mammals can regenerate liver, pancreas and skin, and can partially repair injury of peripheral nerves or skeletal muscle, but retain poor regenerative ability of other organs [66].

While the adult mammalian heart is considered a terminally differentiated organ, the fetal heart retains a remarkable proliferative capacity, capable of regenerating cardiomyocytes following injury [67, 68]. Porrello and colleagues [6] have shown that neonatal mouse cardiomyocytes can regenerate following surgical resection of the apex of the left ventricle. However, by day 7, the regenerative capacity is lost and replaced by a more classical fibrotic response resulting in scar tissue and impaired cardiac function. This finding has re-energized the search for mechanisms that underpin cell cycle arrest in the mammalian heart, with the intention of pursuing key molecular targets that might induce mature mammalian cardiomyocytes to re-enter the cell cycle.

Following transmural myocardial infarction (MI) in adult rats and human, the typical response to injury is inflammation and ventricular remodeling with a fibrotic scar forming at the site of MI [69, 70] (see Table 1). Reparative fibrosis appearing within and around the MI region is essential to safeguard the structural integrity of infarcted tissue, although interstitial fibrosis in non-infarcted myocardium alters tissue stiffness and can lead to ventricular dysfunction [71]. Following such injury, mammalian myocardium typically responds with cardiomyocyte hypertrophy combined with hyperplasia of non-cardiomyocyte cell populations in the heart including fibroblasts [5]. Cardiac hypertrophy, characterized by an increased cardiomyocyte size, enhanced protein synthesis and re-organization of the sarcomere [72], is acknowledged as an adaptive response intended to preserve cardiac function in the face of increasing haemodynamic stress. This physiological response normalizes wall tension and is necessary in the short term to allow the animal to adapt to the initial myocardial loss or haemodynamic load [5]. However, more prolonged stress converts this to a pathological process where a prolonged stimulus to cardiac hypertrophy leads to abnormal cellular responses within the myocardium typified by interstitial fibrosis, a switch to less-efficient myosin types and abnormal calcium handling [73]. These features result in so-called adverse remodeling of the ventricle and, in addition to contractile dysfunction, can lead to congestive cardiac failure and its fatal sequelae of progressive pump failure or sudden arrhythmic death. Apoptosis is also a feature of cardiac hypertrophy particularly when associated with congestive heart failure [74]. However, evidence has emerged that the adult mammalian heart does contain a small population of progenitor cells capable of differentiating into cardiomyocytes [75]. These cells might play a role in the replacement of lost cardiomyocytes [76–78], although they do not appear to be activated as part of a replacement process [59].

**Table 1:** Comparative scheme outlining the cardiac response to injury in mammals versus zebrafish

	Human	Mice	Zebrafish
Response to injury	Fibrosis followed by cardiomyocyte hypertrophy		Cardiomyocyte proliferation
Cardiac injury end-point	Heart failure/contractile dysfunction		Normal functionality re-established
Regenerative potential	Unknown	Up to 7 days after birth in mice	Lifelong

Nevertheless, the presence of active DNA synthesis [35], even if low, and the presence of putative progenitor cells within the hearts of adult mammals, gives rise to the possibility that proliferation of adult cardiomyocytes could be stimulated therapeutically as part of a process that could support repair and recovery. Why existing cardiac progenitor cell populations are unable to repair cardiac muscle in response to injury and, most importantly, how to trigger this untapped resource, are not yet clear.

Currently, many laboratories and institutions around the world are focused on the engraftment of various types of progenitor cells into infarcted hearts to achieve myocardial tissue renewal [79–83]. To date, most of these studies suggest that, while there may be small improvements in cardiac function, there is little evidence, in both human and mouse models, that such progenitors truly engraft and mature into active cardiomyocytes [84, 85]. There is evidence, however, that they may increase angiogenesis, leading many to believe that progenitor cells release a variety of growth factors that contribute to the response to injury by stimulating growth and repair of non-cardiomyocyte-derived cells [86–88].

### The zebrafish

While mammals lose the ability to regenerate the heart within a few weeks after birth, lower vertebrates, such as amphibians and fish, retain this ability to regenerate the heart, and, indeed, most of their organs, following significant injury or loss of tissue well into adulthood (see table). In the zebrafish, resection of up to 20% of the ventricle apex results in complete regeneration and repair of the ventricle within 60 days [89]. Zebrafish heart regeneration proceeds through injury-induced proliferation of cardiomyocytes which retain their capacity to divide and proliferate postnatally [33, 89–91]. BrdU labelling studies have shown BrdU-positive cardiomyocytes along the leading edge of the regenerating heart. Lepilina and co-workers [92] suggested that regenerating myocardium arises and matures from undifferentiated cardiomyocyte progenitor cells of epicardial origin. In contrast, two more recent genetic fate-mapping studies [7, 11] unambiguously demonstrated that pre-existing committed cardiomyocytes are, in fact, the main source of the cells contributing to cardiac regeneration in the zebrafish.

Thus, although cellular mechanisms involved in cardiac regeneration have recently been unraveled, the molecular pathways that might be involved in initiating and maintaining the cardiomyocyte response to injury remain uncertain. Further studies are required to clarify these mechanisms that induce a hyperplastic, and not a hypertrophic, response in this setting. Genetic similarity between humans and zebrafish [93, 94] support the notion that cardiac regenerative pathways can be dissected in the fish model.

## Zebrafish embryo as a model of cardiomyocyte proliferation

The zebrafish heart is emerging as an increasingly flexible model to study many developmental, genetic and acquired cardiac disorders [95]. Zebrafish heart development is well characterized [9, 96–98] adding to numerous early publications on this topic [99, 100]. Despite having only two chambers, the fish heart retains many of the structural and developmental components of the mammalian heart [101], including a three-layer ventricular wall (epicardium, myocardium and endocardium) from three days post fertilization [102, 103]. The early mammalian heart is derived from first and second heart fields [102, 103], although there is evidence that a population of lateral mesoderm-derived haemangioblasts represent an evolutionary antecedent of the second heart field in zebrafish [104].

The zebrafish embryonic heart is particularly suited to studying aspects of cardiomyocyte proliferation for a number of important reasons. First, the embryonic heart in the early stages is composed of only a few hundred cardiomyocytes [105, 106], and this allows accurate and reproducible approaches to count total cardiomyocyte number in either chamber or both. In addition, transgenic technology using fluorescent proteins [107, 108] can be targeted to developing cardiomyocytes, which allows the ready visualization of in vivo development and function of these cells [109].

The small size of the zebrafish embryo permits exchange of gases by passive diffusion, allowing their survival and relatively normal development for several days even in the absence of a functioning heart and circulation [110]. This permits the phenotypic analysis of embryos with severe or lethal cardiovascular mutations and defects [111, 112] which would otherwise be extremely difficult to assess in higher vertebrates where they would be highly likely to die in utero [13].

There are no well-described models of myocardial infarction or cardiac hypertrophy in lower vertebrates akin to those developed and exploited over many years in small mammals [113]. To circumvent this problem, researchers have developed more radical approaches of resecting a piece of the ventricle. This was first reported in salamanders [114] and then in the zebrafish, where approximately 20% of the ventricle apex can be resected, and regrowth will occur within 60 days [89]. This model has now been reproduced in many laboratories around the world and has become a standard approach to studying the molecular and cellular mechanisms associated with cardiomyocyte proliferation leading to what has come to be known as cardiac regeneration. However, this resection model is only feasible in a low-pressure heart such as that found in lower vertebrate species and in the early postnatal time period in mice. In a high-pressure haemodynamic system, typical of adult mammalian hearts, acute bleeding from the resection margin results in rapid death. Not only does this indicate that the injury response to heart resection is different in zebrafish due to the underlying physiology, but it also poses the question of whether repair of a resected piece of heart by cardiomyocyte proliferation is likely to convey an evolutionary advantage for mammals since it would most likely result in exsanguination from the heart itself.

Arguably, therefore, the best option for a mammalian heart, working at higher physiological pressures, and faced with a large zone of injury is to generate an area of scar tissue as quickly as possible in order to heal the infarcted territory and avoid the risk of myocardial rupture and bleeding that would ensue [115]. In addition, since this ventricle apex resection is not as physiologically or clinically relevant as a model [116], others have used cryoinjury in which liquid nitrogen is used to injure a localised region of ventricle in a regional manner similar to myocardial infarction [117, 118]. Following this cryoinjury, the adult zebrafish heart demonstrates features consistent with regeneration including cardiomyocyte proliferation, but in this case, there is also a more readily observed area of scar which requires up to 3 months for complete repair.

Mechanisms controlling organ and tissue regeneration are conserved between larvae and adults at the cellular and molecular levels, suggesting that the regenerative machinery directing cell proliferation in response to injury may exist from early developmental stages [119]. Indeed, amputation of either the adult or embryo zebrafish fin, a widely used model of tissue regeneration, induces proliferation of progenitor cells from the amputation margin [120]. In the same model, at molecular level, transcription factors and components of various signaling pathways normally upregulated during adult fin regeneration [121] are also increased during larval regeneration [119–121].

We have recently developed and validated a model of heart injury and recovery in the zebrafish embryo using highly targeted laser injury [122] (supplementary movie). The use of embryos significantly shortens the injury response and subsequent repair time period. We have demonstrated a striking ability of the zebrafish embryonic heart to regenerate and recover cardiac function by 24 h post-laser injury. Using this approach, the zebrafish embryo can be used in a high-throughput model system to study human disease. For example, figure S1 clearly shows a protocol to study heart response to injury that can be completed in an experiment over a period of only 4–5 days. Embryos can be injected at 1–2 cell stage with one or more compounds, such as labelled mRNA, for overexpressing genes, or CRISPR/Cas 9 for gene knockout or morpholino oligonucleotides for gene knockdown (figure S1A). Otherwise, embryos can be exposed to small molecules and pharmacological compounds of interest by simply adding these to the medium at the desired developmental stage (figure S1B). Embryos can then be injured, for example using a targeted laser at 72 hpf as described, followed by collection of data of cardiac function, including ejection fraction and tail blood flow (figure S1C). Using transgenic lines expressing fluorochrome driven by cardiac markers, such as tg(myl:gfp)^y1^ in figure S1D, hundreds of embryonic hearts can be isolated in a few minutes, for immunohistochemistry, gene and protein analysis (Figure S1E). The laser injury technique also lends itself to a high-throughput approach; it is possible to create a standardised level of heart injury to the ventricle in approximately 50 embryos per hour. We have also applied the laser-induced injury to different cardiac regions and structures in the zebrafish larva, including bulbus arteriosus and atrioventricular valve and have demonstrated the potential of the zebrafish to be used for various models of cardiac disease [123]. Although the overlying tissue, the pericardium in this case, is also partially injured, this is proportionately less compared to existing surgical approaches (apical resection, cryoinjury) in adults requiring damaging tissue layers covering the heart, making our model more myocardial-specific.

Using the laser-induced injury model, we have explored a number of molecular pathways that could enhance recovery from myocardial injury. In particular, we explored the relationships between cyclin-dependent kinase (CDK)9, La-related protein (LARP)7 and the positive transcription elongation factor (P-TEF)b complex of which they are molecular partners. CDK9 has been implicated in mammal cardiac hypertrophy [124]. In our experiments, we first downregulated CDK9 and or LARP7 by pharmacological or morpholino knockdown and found that they had mainly opposite effects, to CDK9 strongly reducing its action and LARP7 slightly increasing cardiomyocyte proliferation [125]. In separate experiments, we found that prior CDK9 downregulation impaired the cardiac recovery from laser injury, whereas LARP7 knockdown did not. Interestingly, co-injection of LARP7 and CDK9-targeted morpholinos rescued the CDK9 phenotype, both in terms of cardiomyocyte proliferation and cardiac function. We concluded that LARP7 acts to maintain CDK9 in an inactive state in the P-TEFb complex and that suppressing LARP7 activity results in a derepression of CDK9 that ultimately leads to a more active P-TEFb complex. Studying the repair process following injury during cardiogenesis and development clearly raises some questions of relevance of these experiments to the mammalian heart. However, compared to the adult, the zebrafish larva not only offers the advantage to study hundreds of animals over a short time period, but it also allows the possibility to study severe developmental cardiac defects which would otherwise be extremely challenging to study in mammalian models. High-throughput drug screening programs, for example, have been successfully used in zebrafish to explore novel therapeutic candidates before moving forward to more expensive mammalian model systems [126–128].

## Molecular pathways linked to cardiomyocyte proliferation as targets for drug development

**Fig. 2:**
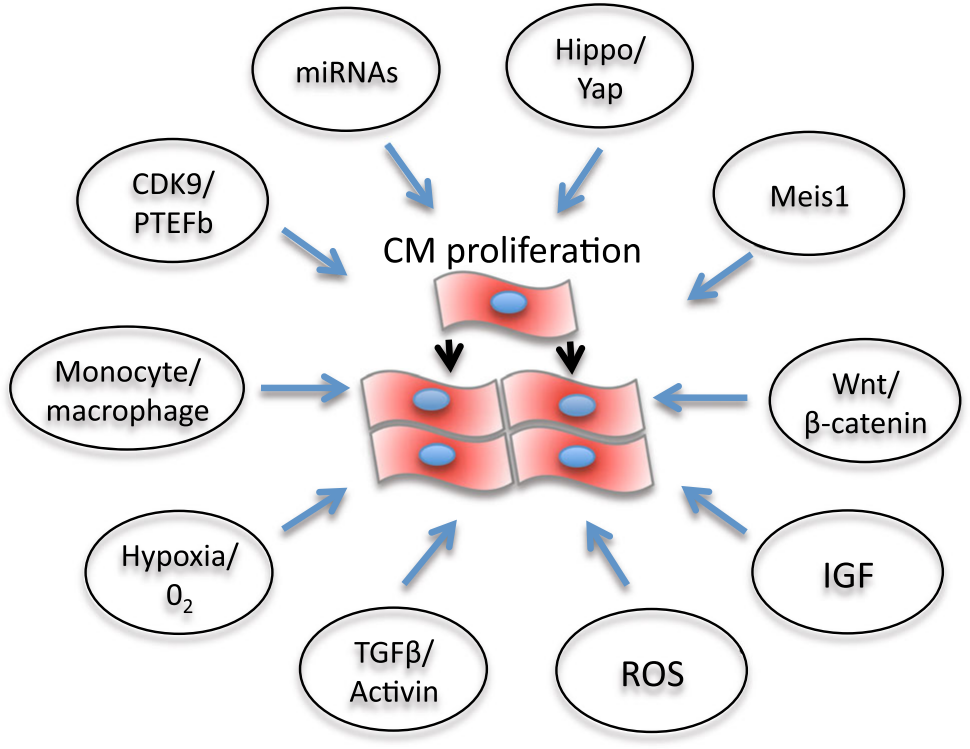
Schematic representation of possible molecular pathways that could be targeted therapeutically to promote cardiomyocyte proliferation in mammals (see text)

Several molecular pathways are under investigation for their potential ability to inhibit or activate cardiomyocyte growth and or proliferation and could be harnessed to develop heart disease therapies (Fig. 2). In some cases, the role of these pathways in cardiomyocyte proliferation has been unraveled in zebrafish. For example, a balance between CDK9 and its repressors, including LA-Related Protein 7, can switch on or off cardiomyocyte proliferation in zebrafish [125]. However, CDK9 knockdown affects somatic growth and development of a number of key embryonic structures including the brain, heart, eye and blood vessels [129]. For therapeutic strategies, this raised the question to develop tissue- or cell-specific anti-CDK9 drugs. The activity of the P-TEFb complex, a key driver for transcription formed by CDK9 and cyclin T, is increased in cardiac hypertrophy in mammals [124]. In addition, the CDK9 activity is derepressed by the dissociation of 7SK small nuclear RNA [130, 131] and HEXIM1 [132, 133], two CDK9 inhibitors. HEXIM1 knockout mice die during fetal development and exhibit all the genetic and physical hallmarks of cardiac hypertrophy [134]. CDK9 has also been shown to regulate cell cycle and is involved in cardiomyocytes differentiation from mice embryonic stem cells [135]. Mahmoud and colleagues [29] showed that in neonatal and adult cardiomyocytes, Meis1 deficiency increases cardiomyocyte numbers, whereas Meis1 overexpression activates CDK inhibitors INK4b-ARFINK4a and Cdkn1a genes, which leads to cell cycle arrest because of inhibition of CDKs. miRNAs are also potential targets for cardiomyocyte proliferation therapies (Fig. 2) [136]. As miRNAs generally repress gene expression by promoting mRNA degradation and/or by inhibiting translation, the final effects on cardiomyocte proliferation are mediated by inhibiting or activating the cell cycle [137, 138]. For example, miR-590 and miR-17/92 clusters promote cardiomyocyte proliferation by inhibiting the proliferation repressors Homer protein homolog 1 (Homer1) and Homeodomain-only protein x (Hopx), whereas miR-15 family represses cardiomyocyte proliferation by inhibiting the proliferative activator Checkpoint kinase 1 (Check1). Furthermore, the muscle-specific microRNA-1 (miR-1), which normally keeps CDK9 derepressed at the transcriptional level [139], was downregulated at a very early stage, following cardiac hypertrophy induced in a mouse model of aortic constriction-induced hypertrophy.

Also, the Hippo/Yap pathway plays an essential role in the regulation of heart development and postnatal cardiomyocyte proliferation [140], highlighting the potential for enhancing cardiac regeneration (Fig. 2) [141]. Indeed, modulation of this pathway in the neonatal heart has been shown to prolong the regenerative window. However, Hippo/Yap also appears to influence cardiomyocyte autophagy and apoptosis [142]. Among the mitogens, IGF2 has been shown to activate cardiomyocyte proliferation and is required for zebrafish heart regeneration [143], whereas TGFβ/activin signaling plays important roles in cardiomyocyte proliferation and scar formation [144].

Modulation of inflammation may provide a key therapeutic strategy to drive heart regeneration. Indeed, the types of macrophage present in the regenerating neonatal mouse heart may provide essential stimuli for angiogenesis and regeneration [145].

Hypoxia, redox signaling and metabolic phenotypes are also key regulators of cardiomyocyte proliferation and cardiac renewal [146]. Puente et al. [147] have recently shown that cell cycle arrest in postnatally terminally differentiated cardiomyocytes is triggered by mitochondrial reactive oxygen species-mediated oxidative DNA damage. This suggests that ROS may play key roles in cell cycle regulation and differentiation during cardiac development. In turn, this suggests that cells responsible for cellular turnover in the heart, such as immature and/or mature myocytes or progenitor population may need an environment with a lower oxygen concentration to proliferate efficiently. The heart may be unique in the nature of this oxygen-sensitive response. On the one hand, it has high O_2_ consumption [148], and on the other hand, its cardiac progenitor cells appear to benefit from hypoxic preconditioning which improves survival and homing of engrafted cells into an infarcted territory [149]. Indeed, the epicardium and subepicardium regions contain multipotent progenitor cells [150] which could represent a novel hypoxic niche of the heart. These cells express Hif1α and respond to hypoxia by increasing cell proliferation and, thus, provide a source of new cardiac cells following injury including fibroblasts, perivascular smooth muscle cells [151] and cardiomyocytes after thymosin β4 activation [144, 145, 152]. A better understanding of these and other molecular mechanisms would allow us to develop exciting new strategies to improve cardiomyocyte proliferation and cardiac regeneration.

## Future perspectives

The question remains as to how drugs or therapeutic stategies could be used to support cardiac repair in disease conditions where the heart was failing or at risk of failing following injury, stress or cell loss. Clearly, a key time for treatment would be around the time of an acute myocardial infarction where many previous studies of bone-marrow-derived stem cells have been extensively tested over the last 10 years [153] with overall, relatively disappointing results.

The possibility of directly harnessing cellular mechanisms to achieve therapeutic benefit in patients with both heart disease remains elusive. To date, the possibility of providing therapies targeted at enhancing cardiomyocyte proliferation that could either be administered acutely, at the time of acute injury such as infarction, or in the long term to patients with chronic left ventricular systolic dysfunction remains an important but elusive target. The key issue would be whether such drugs could be administered orally or intravenously and whether their benefits outweighed the risk of harm. At this stage, we are a long way from knowing the answers to these questions since no viable candidate drug has yet been developed despite a number of interesting pathways currently undergoing further studies. Any such drug with the capacity to reactivate a highly differentiated cardiomyocyte into a more active proliferative state is likely to carry a significant risk of inducing unregulated cell growth either in the target tissue or in other exposed tissues. The risk of neoplasia is, therefore, clear unless the drug can be highly targeted to a specific cardiac pathway or delivered in a highly localised manner directly to the heart.

### Electronic supplementary material

Below is the link to the electronic supplementary material.

Supplemental movie - Laser pulse injury of the zebrafish embryonic heart **–** A single laser pulse, in this example at the atrioventricular cushion of a zebrafish embryo at 72hpf, produces an instantaneous cessation of ventricular contraction and gradual recovery of cardiac rhythm over the next few minutes. Laser ablation also results in regurgitation of blood from ventricle to the atrium resulting in flow reversal in the cardinal veins (MOV 1712 kb)

Figure S1- Scheme outlining the efficacy of zebrafish embryo as an ideal model for a high throughput approach. A. Zebrafish genome can be manipulated by injection at 1-2 cell stage eggs of molecular compounds such as CRISPR/Cas9 or morpholino. In this images, a morpholino tagged with lissamine as tracker was injected and visible under fluorescence light. B. Zebrafish larvae, here at 48hpf, can be exposed to drug and/or the hearts can be injured by laser. C. Cardiac function can be easily assessed later during the development by ejection fraction or tail blood flow analysis. D. Many zebrafish larvae hearts can be easily isolated in a few minutes from a tg(*myl7:gfp*)^y1^ line and be used for several applications, including immunostaining, gene and protein expression. (The content in this figure is responsibility of the authors) (PDF 1394 kb)
