# Supplementary material for: Cardiomyocyte proliferation in zebrafish and mammals: lessons for human disease
Source: Cell Mol Life Sci. 2016 Nov 3;74(8):1367–78. doi: 10.1007/s00018-016-2404-x (PMC5357290; doi:10.1007/s00018-016-2404-x)
Supplement: Supplementary file 2 — Figure S1- Scheme outlining the efficacy of zebrafish embryo as an ideal model for a high throughput approach. A. Zebrafish genome can be manipulated by injection at 1-2 cell stage eggs of molecular compounds such as CRISPR/Cas9 or morpholino. In this images, a morpholino tagged with lissamine as tracker was injected and visible under fluorescence light. B. Zebrafish larvae, here at 48hpf, can be exposed to drug and/or the hearts can be injured by laser. C. Cardiac function can be easily assessed later during the development by ejection fraction or tail blood flow analysis. D. Many zebrafish larvae hearts can be easily isolated in a few minutes from a tg(myl7:gfp)y1 line and be used for several applications, including immunostaining, gene and protein expression. (The content in this figure is responsibility of the authors) (PDF 1394 kb) [file 18_2016_2404_MOESM2_ESM.pdf]

Figure 1

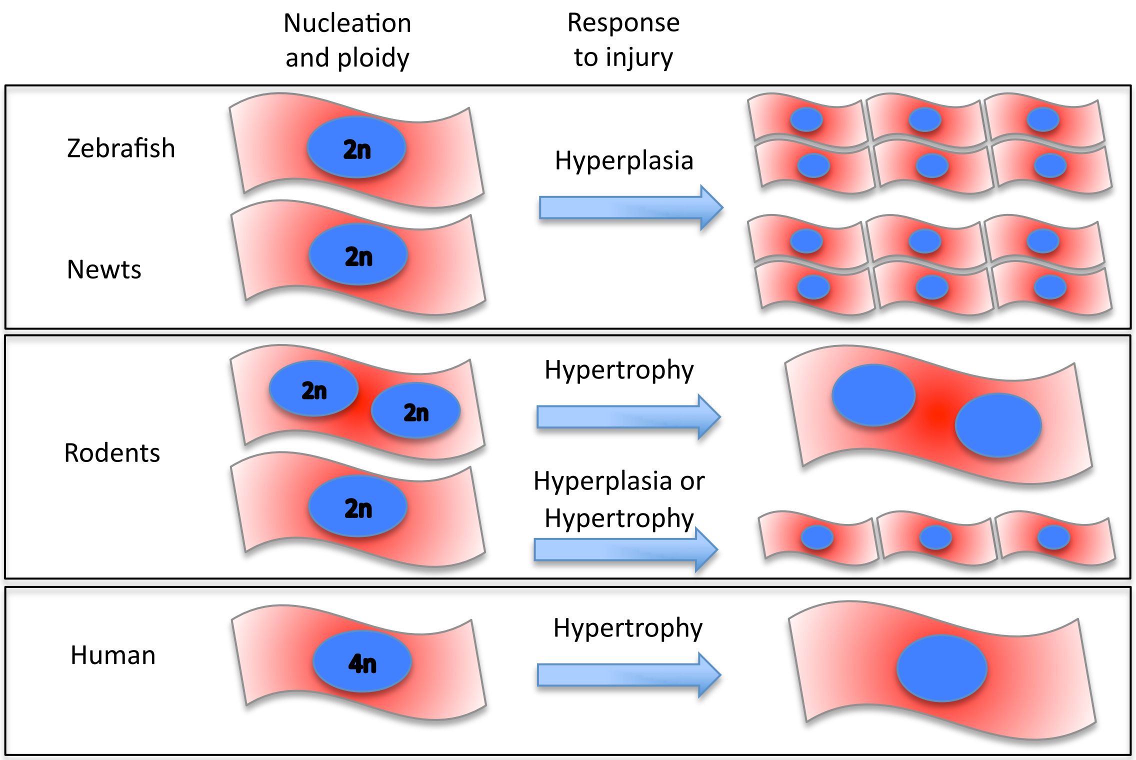

Nucleation and ploidy
Response to injury
Zebrafish
Newts
2n
2n
Hyperplasia
Rodents
2n
2n
2n
Hypertrophy
Hyperplasia or Hypertrophy
Human
4n
Hypertrophy

Table - Comparative scheme of cardiac response to injury in Mammals and Zebrafish.

	Human	Mice	Zebrafish
Response to injury	Fibrosis followed by cardiomyocyte hypertrophy		Cardiomyocyte proliferation
Cardiac injury end-point	Heart failure/Contractile dysfunction		Normal functionality re-established
Regenerative potential	Unknown	Up to 7 days after birth in mice	Lifelong

Figure 2

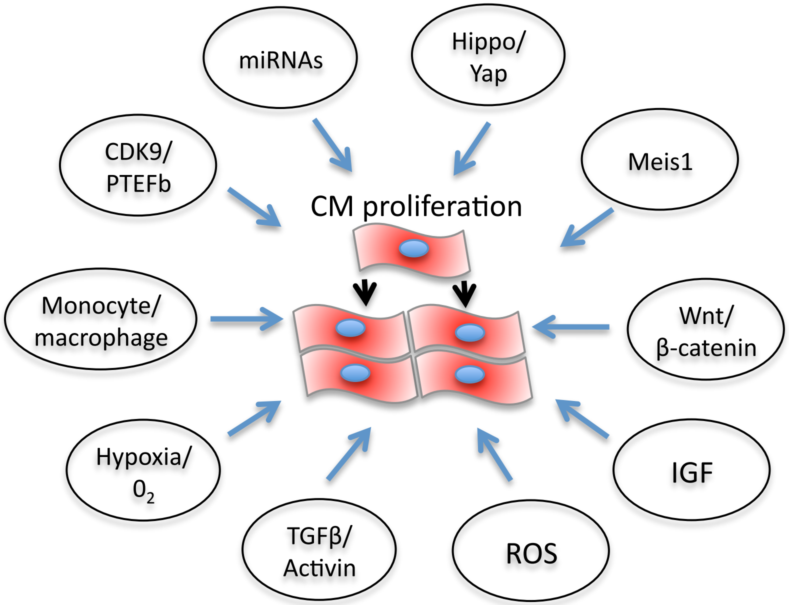
miRNAs
Hippo/
Yap
CDK9/
PTEFb
Meis1
CM proliferation
Monocyte/
macrophage
Wnt/
β-catenin
Hypoxia/
0_2
IGF
TGFβ/
Activin
ROS

Figure S1

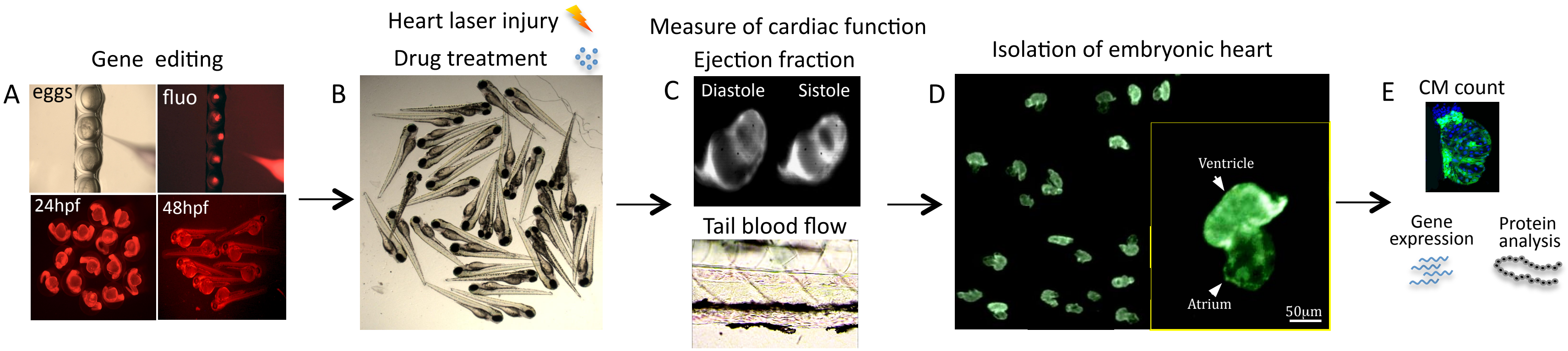
Gene editing
A
eggs
fluo
24hpf
48hpf
Heart laser injury
Drug treatment
B
Measure of cardiac function
Ejection fraction
C
Diastole
Sistole
Tail blood flow
Isolation of embryonic heart
D
Ventricle
Atrium
50µm
E
CM count
Gene expression
Protein analysis
